# The Inverse Association of Mediterranean Diet with Emotional Eating: A Cross-Sectional Study in Greek Adults

**DOI:** 10.3390/diseases13050151

**Published:** 2025-05-14

**Authors:** Maria Mentzelou, Sousana K. Papadopoulou, Aikaterini Louka, Georgia-Eirini Deligiannidou, Evmorfia Psara, Constantinos Giaginis

**Affiliations:** 1 Department of Food Science and Nutrition, School of the Environment, University of the Aegean, 81400 Myrina, Lemnos, Greece; maria.mentzelou@hotmail.com (M.M.); loukathy612@gmail.com (A.L.); fnsd21013@fns.aegean.gr (E.P.); 2 Department of Nutritional Sciences and Dietetics, School of Health Sciences, International Hellenic University, 57400 Thessaloniki, Greece; deligiannidoueirini@yahoo.gr

**Keywords:** emotional eating, emotional food consumption, MedDiet score, Mediterranean diet, dietary pattern, eating behavior, three-factor eating questionnaire

## Abstract

Background/Objectives: Emotional eating (EE) is the tendency to overeat in response to negative emotions. Food consumption is influenced by both personal and environmental factors. Emotions are personal factors that can affect food consumption. The objective of this study is to assess the association between Mediterranean diet (MD), a dietary pattern promoting mental health, and emotional eating via the Three-Factor Eating Questionnaire (TEFQ). Methods: This is a cross-sectional survey including 328 adults aged 18–75 years. Appropriate questionnaires were applied for evaluating adherence to the Mediterranean diet (MedDiet score) and types of feeding and the expression of emotional food consumption (TFEQ). Results: A mean MedDiet score equal to 30.97 ± 4.93 and a total TFEQ score equal to 45.40 ± 6.31 were noted. MedDiet score was significantly inversely associated with TFEQ total score (r = 0.23, *p* = 0.026) as well as with TFEQ emotional score (r = 0.37, *p* < 0.0001). Fruits and vegetables consumption was positively associated with TFEQ emotional score (r = 0.25, *p* = 0.014, and r = 0.20, *p* = 0.049, respectively). Conclusions: In order to produce data showing improvements in eating behavior, our findings have highlighted the significance of conducting large, prospective, well-designed, randomized, interventional clinical studies to confirm the inverse association of MD with EE. The interpretation of the results is complicated due to cross-sectional design, the social desirability bias and the self-report nature of both dietary and emotional assessments.

## 1. Introduction

Emotional eating (EE), which can be triggered by positive feelings, is the need to eat in order to suppress, dull, or manage strong negative emotions including stress, anxiety, loneliness, anger, or grief. Since stress is viewed as undesirable, eating becomes a coping mechanism for adverse emotional states, and EE is a habit defined by the inability to differentiate between physiological feelings of hunger and negative emotions [1,2]. The two-way interaction between emotional triggers and weight fluctuations creates a vicious loop that makes treating EE as a dysfunctional eating behavior challenging [3].

Several studies have demonstrated that while modified emotion regulation techniques can assist people deal with stressful situations and defend against negative feelings, a wide range of medical and psychological issues have been linked to emotion management failures. Furthermore, those who have dysregulated emotions may engage in impulsive eating, which is the act of eating in reaction to unpleasant emotions [4]. Emotional dysregulation connects pathologic eating behaviors and psychopathologic traits in bariatric surgery candidates [5].

Food consumption fluctuations can be explained by changes in specific aspects of eating behavior, including EE, which is the desire to eat in response to feelings rather than physical hunger, Cognitive Restraint (CR), which is an attempt to limit food intake, and Uncontrolled Eating (UE), which is a loss of control in the presence of palatable stimuli. In this respect, the three-factor eating questionnaire (TFEQ-R18) can be used to measure the above behaviors. Thus, as a popular tool for measuring behaviors linked to the development of obesity, the TFEQ-R18 has been validated for use with a number of populations, including adolescents, general adults, women in their midlife and beyond, women with gestational diabetes, and adults who are overweight or obese [6].

Emotions are widely recognized to play a significant role in food elections [7]. Both the amount and quality of food consumed can be influenced by emotional moods. According to studies done in the last few decades, eating more is frequently linked to stress and harmful emotions, unless the person is experiencing extreme emotional states like anxiety or panic, in which case appetite may completely vanish [8,9,10]. As a coping mechanism, anxiety can also increase the intake of foods heavy in fat and sugar. It can also change the brain’s reward system, which can lead to a shift from healthy eating patterns to unhealthy ones. Consuming “comfort food”, or meals rich in calorie density, like sweet, high-fat foods, is common among those with depressed symptoms [11]. Moreover, a higher energy-dense diet was linked to a 2.56-fold higher incidence of stress in a prior study [7].

Researchers should be able to assess EE in order to investigate it. Several self-report EE surveys have been created for this reason [12,13]. One instrument for evaluating eating habits is the TFEQ. It was first created in 1985 by Stunkard and his associates, and it has subsequently undergone revisions and modifications [14]. Three primary aspects of eating behavior are examined by the TFEQ: (a) Cognitive constraint is the degree to which an individual limits their food consumption in order to manage their weight. Avoiding overeating and making healthier choices even when one is hungry are examples of cognitive restraint; (b) Disinhibition is the inability to regulate one’s food intake in reaction to emotional or environmental cues. People with strong disinhibition often overeat when under stress or in the presence of appetizing foods; and (c) The subjective sensation of hunger and the propensity to consume more when experiencing hunger are referred to as hunger.

A plant-based diet that is well-balanced is the Mediterranean diet (MD). Several studies have demonstrated the health benefits of the MD for people who adopt and adhere to this healthy eating pattern. These advantages stem from the food’s quality, preparation, and nutritional content in addition to its type. In addition to consuming a diet low in animal fats and refined carbohydrates, this dietary model is distinguished by the consumption of fresh, seasonal, and local foodstuffs [15]. However, because of the differences in culture, religion, ethnicity, and socioeconomic level across and within the many Mediterranean countries, its precise meaning varies slightly [16]. However, a MD is typically defined by modest intakes of fish, chicken, potatoes, legumes, eggs, and sweets, as well as frequent consumption of non-refined grains, vegetables, fruits, nuts, olive oil, and dairy products. Lastly, two key elements of the Mediterranean lifestyle are increased physical activity and a moderate consumption of red wine during meals [17]. Notably, MD adherence has highly been associated with a lower risk of morbidity and mortality, acting as a preventing factor against several chronic diseases, including mental health disorders [15,16,17]. Additionally, the MD balanced approach to nutrition contributes to the establishment of healthy eating habits that can last a lifetime, promoting long-term well-being [15,16,17].

MD constitutes a traditional healthy dietary regime for the population of Mediterranean countries, including Greece. Since EE is associated with consuming high-calorie foods and MD is characterized by low consumption of energy dense foods, while also being associated with improved mental health [18], the aim of the present study was to examine for the first time the relationship between MD and EE through the TFEQ in a Greek adult population.

## 2. Methods

### 2.1. Design and Sample

This is a cross-sectional survey with 328 adults. Participants were randomly selected, and the selection was restricted to individuals who met specific criteria like the age range (18–75 years) and the absence of any chronic disease such as cardiovascular diseases, metabolic disorders, cancer, mental health disorders (e.g., depression, anxiety, stress, etc.), or eating disorders such as anorexia nervosa and bulimia nervosa. All participants’ data remained entirely confidential. The enrolled adults were informed concerning the aims of the current survey, as well as the confidentiality of their data, and approved to voluntarily take part in this survey. SPSS software version 29.0.2.0 was used to perform power analysis and sample size calculations. The sample size calculation suggested a number of approximately 300–350 participants as adequate for this study. The statistical power analysis of the study was estimated to be equal to 84.3%. The survey was performed in agreement with the Declaration of Helsinki and approved by the Institutional Ethics Committee of the University of the Aegean (protocol code 1218/12.4.2022 and date of approval: 12 April 2022).

### 2.2. Adherence to the Mediterranean Diet

Panagiotakos et al. created and described MedDiet score adherence by assigning intake of the goods contained in the MedDiet a score ranging from 0 to 5, where the latter indicates the highest adherence. The overall score varies from 0 to 55 points. The consumption of potatoes was given a special category with a score of five points for consuming three to four portions per week, four points for consuming one to two portions per week, and lower scores for consuming potatoes on a weekly or daily basis. Using wine glasses as a reference, alcohol consumption was also taken into account. Drinking less than three glasses of wine per day earned a maximum score of five points; drinking more than seven glasses earned no points; and consuming three to seven glasses of wine per day earned scores ranging from one to four [19].

### 2.3. Emotional Eating Evaluation

All participants’ questionnaires were completed through one-to-one interviews in a calm atmosphere to respect the confidentiality of the procedure. To assess types of feeding and the expression of emotional food intake in response to emotion, a weighted Greek version questionnaire for adults was utilized: the Greek version of TFEQ [20]. The TFEQ comes in a number of variations, including the TFEQ-R18 (18 items), which modifies and streamlines the original survey for use with varied demographics and objectives [14]. At the same time, it also evaluates types of uncontrolled and restrictive eating behaviors. Cronbach’s alpha, an index of TFEQ reliability, was estimated to be 0.81.

### 2.4. Statistical Analysis

Extensive tests were applied for evaluating the associations between TFEQ emotional score and demographic and anthropometric parameters. Continuous variables were expressed through mean and standard deviation (SD), median, and minimum and maximum scores. Categorical variables were expressed as absolute frequencies and relative frequencies on a percentage setting (%). The Kolmogorov–Smirnov (K-S) test and histogram analysis were applied for assessing the normality of distribution of continuous variables. The Pearson correlation coefficient was used to assess the correlations of MedDiet score and its components with total TFEQ and its dimensions. Univariate regression analysis was performed based on one-way ANOVA. The level of statistical significance was set at α = 5%. SPSS software version 29.0.2.0(20) was used for the analyses.

## 3. Results

### 3.1. Descriptive Statistics of the Study Population

The descriptive data included information concerning the sample distribution regarding gender, employment and marital status, smoking habits, educational level, and age (Table 1). Table 1 includes the sociodemographic parameters of the study population (n = 328 adults). The mean age of the enrolled population was 36.4 ± 8.2 years. Regarding gender, 82.31% of the sample consisted of women (270 people). Regarding the working status of the participants, 82.62% reported that they were employed (n = 271), while 56.70% reported that they were single (n = 186). Concerning smoking habits, 85.67% of the enrolled adults were non-smokers (281 people), whereas concerning educational level, 47.26% were university graduates (155 people) and 32.62% held a master’s degree (107 people). We also performed a multicollinearity process between all the variables, and we did not find any correlation amongst the examined variables.

In Table 2, regarding the total MedDiet score, an average of 32.97 (standard deviation 4.93) is observed. The median is 33.00, while the minimum value is 18.00 and the maximum is 51.00. Concerning the TFEQ Uncontrolled variable in Table 3, the mean value is 23.4 (SD: 4.01), and the median is 23.00 (minimum–maximum values: 2.00–34.00). On TFEQ Cognitive Restraint, the mean value is 15.06 (SD: 2.26), and the median is 15.00 (minimum–maximum value: 8.00–22.00). Concerning the total TFEQ score, the mean value is 45.40 (SD: 6.41). The median is 45.00 (minimum–maximum value: 21.00–67.00). In TFEQ Emotional, the mean value is 6.94 (SD: 2.6), and the median is 7.00 (minimum–maximum value: 3.00–12.00).

### 3.2. Regression Analysis for the Correlation of TFEQ Emotional

Table 3 includes the regression findings concerning the correlation of the TFEQ Emotional as dependent variable with MedDiet score after adjustment for potential confounders such as age, gender, working condition, marital status, smoking habits and educational level. None of the confounding factors were significant. In regression analysis, the coefficient β1 is −0.413 with a standard error of 0.240, and the coefficient β2 (related to the MedDiet Score) is 0.008 with a standard error of 0.004. The *p*-value of the model is 0.005, indicating statistical significance, and the adjusted R^2^ is 0.040, which shows a relatively small contribution of adherence to the MD in explaining the variation.

Table 4 includes the findings of the correlation of the TFEQ Emotional score with MedDiet score. The table shows that statistically significant differences exist in the distribution of individuals in total MedDiet score, and these were significantly associated with TFEQ total score (r = 0.23, *p* = 0.026) as well as with TFEQ emotional score (r = 0.37, *p* < 0.0001). Fruits consumption was associated with TFEQ total score (r = 0.22, *p* = 0.0029). Moreover, fruit consumption was significantly associated with TFEQ cognitive restraint score and TFEQ emotional score (r = 0.23, *p* = 0.024 and r = 0.25, *p* = 0.014). Vegetable consumption was associated with TFEQ emotional score (r = 0.20, *p* = 0.049). A trend of a correlation between vegetable consumption and TFEQ total score was also noted (r = 0.19, *p* = 0.070). In addition, wine consumption was associated with TFEQ emotional score (r = 0.24, *p* = 0.018).

## 4. Discussion

Complex biopsychosocial elements, such as personal psychological profiles, societal settings, and environmental triggers, all have an impact on EE [3]. This study was conducted to explore the relationship between MD adherence and EE among adult individuals. The findings provide new insights into how Mediterranean dietary patterns can regulate EE behaviors. MD adherence was inversely associated with EE, whereas fruit, vegetable, and wine consumption were positively associated with EE.

The relationship between EE and energy, macronutrient intake, or specific meal choices has been evaluated in a number of research papers. Fast food consumption, salty snacks, sweet, high-fat foods, or foods high in energy, such as cakes, biscuits, pastries, ice cream, chocolate and its derivatives, breakfast cereals, candies, and artificially sweetened drinks, have all been linked to EE [21,22]. All the above foodstuffs are avoided when a person adopts the MD. To the best of our knowledge, the present study is the first to investigate EE and adherence to the MD and its components in adulthood.

The MedDiet score is the score that evaluates the degree of adherence to the MD, rated on a scale from 0 to 55. Therefore, our result of 32.97 is an average value. For the inverse relationship observed between the EE score and the MedDiet score up to 32.97 units, it is likely explained by the positive benefits that adopting the MD brings to mental health [23]. However, regarding the positive correlation found for higher MedDiet score, the self-regulatory actions required to achieve even greater adoption may be emotionally exhausting and, at a later stage, ultimately lead to a decrease in dietary self-efficacy [24]. The hedonic pleasure and immediate reward that these appetizing foods offer can divert attention from the experience of unpleasant emotions [25]. Moreover, the cross-sectional nature of the data precludes causal inference.

It should be noted that Buja et al. found that emotional undereating was inversely associated with a good adherence to the MD, whereas no such association emerged for emotional overeating [25]. However, this study was performed in children aged 9–11 years and did not include adults. Moreover, its sample size was quite small (n = 178) compared to our sample size [25]. Several surveys performed on adults and on 12- to 15-year-olds reported that EE was related to the intake of sweet and high-fat foodstuffs, which are avoided in MD [26,27,28]. However, there was also no association indicated between EE and the intake of snacks, sweet foods, or fatty foods in children at the age of 5–12 years [29,30]. Thus, the currently available evidence seems quite conflicting and may be affected by the age of the enrolled individuals, highlighting the demand for future studies in each age stage separately. Moreover, it should be emphasized that the consumption of such foods can substitute for regular meals, subsequently resulting in incorporation of such foods into people’s everyday diet [31]. In addition, Madali et al. showed that during COVID-19 pandemic, there was a rise in the intake of fresh fruits and vegetables, protein sources such as eggs, milk, and red meat, whereas there was a reduction in the consumption of junk food like biscuits, chips, chocolates, and carbohydrates like pastries, syrupy desserts, and bread, which was ascribed to the excess of easily accessible fresh vegetables and fruits [32].

Only one of those studies conducted on a high sample of 4316 adolescents found a significant negative connection between a healthy dietary pattern, which has several similarities with the MD, and EE score. Whole grains, fresh vegetables, fruit, milk, soy products, pork/beef meat, and poultry made up the majority of this dietary pattern [33]. In contrast, there was no significant correlation between EE and a Mediterranean-type pattern in another other study [11]. However, the above study included a lower number of young adults (n = 252 university students) [11]. A systematic review suggested that at any time, food is eaten for the pleasure it produces, or for its taste, but also in search of the feelings of satisfaction or pleasure when consuming it. In this way, a negative emotional state before eating food can be disguised or made more persistent [34]. Moreover, several fruits and vegetables as well as wine are very tasty, and they may cover the hedonic pleasure and immediate reward that these appetizing foods can in terms of diverting attention from the experience of unpleasant emotions [25].

An increasing number of surveys have been published in the previous few years highlighting the beneficial health effects of the MD against several chronic diseases in both Mediterranean and non-Mediterranean populations, decreasing cardiometabolic risk, reducing the risk of diabetes and metabolic-related conditions, preventing some cancer types, and resulting in a lower risk of mental disorders such as cognitive decline and depression [35]. MD was related to lower mean heart rate, a mitigation of the detrimental impacts of overweight/obesity on the risk of cardiovascular diseases, and a reduction of the impacts of obesity on type 2 diabetes, and it can also increase fertility [36,37]. In addition, accumulating evidence shows that the five most crucial adaptations made by the Mediterranean dietary pattern are: (a) lipid-lowering impact, (b) prevention against oxidative stress, inflammation, and platelet aggregation, (c) regulation of hormones and growth factors participated in caner pathogenesis, (d) inhibition of nutrient sensing pathways through specific amino acid restriction, and (e) gut microbiome-facilitated production of metabolites affecting metabolic health [38]. Pammer et al. also showed that a balanced diet in combination with meticulous insulin management could improve multiple metrics of HDL function [39]. Moreover, MD lowered the rate of gestational diabetes mellitus and postpartum metabolic syndrome in women affected by overweight or obesity, supporting evidence that its implementation could be routinely recommended from the initial gestational weeks [40].

Hindi et al. showed that with modest intake of nutrient-dense foods and the MD popularity, targeted interventions and educational policies could encourage healthy nutritional habits, being beneficial for overall public health [41]. Additionally, the relatively low level of adverse environmental effects (water, nitrogen, and carbon footprint) related to the MD constitute a supplementary helpful characteristic of the MD model. It is probable that a healthy diet in combination with the social behaviors and lifestyle of Mediterranean areas make the MD a sustainable lifestyle model that could expected to be adopted in other areas with country-specific and culturally appropriate variations [42]. In the context of a “Planeterranean” framework and perspective, which could be implemented in any European area and worldwide, MD constitutes a healthy and sustainable lifestyle model, being capable of preventing several diseases and decreasing premature mortality. Moreover, the availability of a front of pack (FOP) labeling, like Med-Index, could foster more conscious food choices amongst consumers, focusing their attention both to human and planetary health [43].

## 5. Strengths and Limitations

The present survey has several strengths. The research utilized the validated Greek version of TFEQ, and one-to-one interviewing to minimize recall bias. In addition, there are no adequate data and no interventional surveys for Greek population in the international scientific literature. There is also no available data on the adult population in this field, rendering the present study as the first conducted on an adult population. There are also some limitations to this survey. The cross-sectional design of this survey is a limitation; the results cannot be generalized and cannot support causality effects. Moreover, it was found that when people are in a non-emotionally active phase, they underestimate the effects of emotions on their behavior. Another limitation constitutes the nature of recall bias or social desirability affecting answers, especially in emotional and dietary assessments. We have to mention that most of the enrolled participants were women, which is an additional bias. The findings may be generalized; however, the incidence of EE in Greece remains unknown. In addition, no dietary evaluation was performed. Moreover, we should emphasize that there is a likelihood of the presence of unmeasured confounders, like sociodemographic status, depression, anxiety and stress, eating disorders with psychiatric backgrounds, sleep disorders, and low physical activity levels of the enrolled participants despite our methodical efforts to perform adjustments for confounders. Hence, although we adjusted for multiple confounding factors, it is likely that residual confounding may have influenced our results. Lastly, even though we carried out face-to-face interviews between participants and qualified personnel, self-reported data may include recall bias.

## 6. Conclusions

The present study examined the association between MD and EE behavior. The present study provided novel evidence supporting the currently existing literature with regard to the reverse relationship between EE and MD in adults. Our results provide evidence of the importance of performing large, prospective, well-designed, randomized, interventional, clinical trials to generate data highlighting the interconnections between Mediterranean dietary patterns and EE improvements in eating behavior. Additionally, mental health therapies and integrative health policies should be developed and applied for the evaluation of the prevalence of EE in population-based settings and its associations with dietary patterns that may positively affect the quality of life of the target group. The above novel evidence in the field enriched by prospective studies could offer specific recommendations for practical applications and suggestions for future research on how we could treat emotional eating by adopting a healthy dietary pattern such as the MD.

## Figures and Tables

**Table 1:** Descriptive statistics of sociodemographic characteristics.

**Age (mean, years** **± SD)**	36.4 ± 8.2	
**Gender (Ν, %)**		
**Men**	58	17.68%
**Women**	270	82.31%
**Working condition (N, %)**		
**Unemployed**	57	17.38%
**Employe** **d**	271	82.62%
**Marital status (N, %)**		
**Married**	142	43.29%
**Single**	186	56.70%
**Smoking habits (N, %)**		
**Non** **-** **smokers**	281	85.67%
**Smokers**	47	14.33%
**Education level (N, %)**		
**Secondary education**	49	14.94%
**Universit** **y studies**	155	47.26%
**Master’s degree**	107	32.62%
**PhD degree**	17	5.18%

**Table 2:** MedDiet Score and TFEQ results in the study population.

MedDiet and TFEQ	Mean	S.D.	Median	Min.	Max.
**MedDiet score**	32.97	4.93	33.00	18.00	51.00
**TFEQ Uncontrolled**	23.40	4.01	23.00	2.00	34.00
**TFEQ Cognitive Restraint**	15.06	2.26	15.00	8.00	22.00
**TFEQ Emotional**	6.94	2.60	7.00	3.00	12.00
**Total TFEQ**	45.40	6.31	45.00	21.00	67.00

**Table 3:** Results of regression analysis for correlation of MedDiet score with TFEQ emotional eating.

	Β1 (Standard Error)	Β2 (Standard Error)	*p*-Value	R^2^ Adjusted
MedDiet Score	−0.413 (0.240)	0.008 (0.004)	<0.001	0.040

**Table 4:** Correlation of TFEQ scores with MedDiet score components.

Med Diet Question	TFEQ Total r (*p*)	TFEQ Uncontrolled r (*p*)	TFEQ Cognitive Restrain r (*p*)	TFEQ Emotional r (*p*)
Olive Oil	0.15 (0.156)	0.07 (0.488)	0.07 (0.472)	0.14 (0.173)
Legumes	0.06 (0.572)	−0.02 (0.842)	0.08 (0.445)	0.07 (0.480)
Whole Grains/Bread	0.13 (0.210)	−0.03 (0.806)	0.14 (0.168)	0.17 (0.108)
Fruits	**0.22 (0.029)**	−0.01 (0.958)	**0.23 (0.024)**	**0.25 (0.014)**
Vegetables	**0.19 (0.070)**	0.03 (0.763)	0.15 (0.141)	**0.20 (0.049)**
Fish	0.09 (0.407)	0.05 (0.657)	−0.03 (0.791)	0.14 (0.177)
Meat and Meat Products	0.06 (0.566)	0.11 (0.310)	0.06 (0.590)	−0.06 (0.584)
Poultry products	0.14 (0.163)	0.16 (0.129)	−0.04 (0.695)	0.13 (0.195)
Dairy products	−0.04 (0.674)	0.07 (0.485)	−0.03 (0.742)	−0.15 (0.160)
Wine	0.15 (0.143)	0.13 (0.203)	−0.11 (0.275)	**0.24 (0.018)**
Saturated/Unsaturated Fat Ratio	−0.01 (0.901)	0.00 (0.994)	−0.10 (0.346)	0.06 (0.564)

## Data Availability

Data are available upon request to the corresponding author.
